# Plasma phospholipid pentadecanoic acid, EPA, and DHA, and the frequency of dairy and fish product intake in young children

**DOI:** 10.3402/fnr.v60.31933

**Published:** 2016-08-16

**Authors:** Nicolai A. Lund-Blix, Kjersti S. Rønningen, Håkon Bøås, German Tapia, Lene F. Andersen

**Affiliations:** 1 Department of Pediatric Research, Oslo University Hospital, Rikshospitalet, Oslo, Norway; 2 Department of Nutrition, Institute of Basic Medical Sciences, University of Oslo, Oslo, Norway; 3 Division of Epidemiology, Norwegian Institute of Public Health, Oslo, Norway

**Keywords:** biomarkers, dietary assessment, food frequency questionnaire, fatty acids, plasma phospholipids

## Abstract

**Background:**

There is a lack of studies comparing dietary assessment methods with the biomarkers of fatty acids in children.

**Objective:**

The objective was to evaluate the suitability of a food frequency questionnaire (FFQ) to rank young children according to their intake of dairy and fish products by comparing food frequency estimates to the plasma phospholipid fatty acids pentadecanoic acid, eicosapentaenoic acid (EPA), and docosahexaenoic acid (DHA).

**Design:**

Cross-sectional data for the present study were derived from the prospective cohort ‘Environmental Triggers of Type 1 Diabetes Study’. Infants were recruited from the Norwegian general population during 2001–2007. One hundred and ten (age 3–10 years) children had sufficient volumes of plasma and FFQ filled in within 2 months from blood sampling and were included in this evaluation study. The quantitative determination of plasma phospholipid fatty acids was done by fatty acid methyl ester analysis. The association between the frequency of dairy and fish product intake and the plasma phospholipid fatty acids was assessed by a Spearman correlation analysis and by investigating whether participants were classified into the same quartiles of distribution.

**Results:**

Significant correlations were found between pentadecanoic acid and the intake frequency of total dairy products (*r*=0.29), total fat dairy products (*r*=0.39), and cheese products (*r*=0.36). EPA and DHA were significantly correlated with the intake frequency of oily fish (*r*=0.26 and 0.37, respectively) and cod liver/fish oil supplements (*r*=0.47 for EPA and *r*=0.50 DHA). To a large extent, the FFQ was able to classify individuals into the same quartile as the relevant fatty acid biomarker.

**Conclusions:**

The present study suggests that, when using the plasma phospholipid fatty acids pentadecanoic acid, EPA, and DHA as biomarkers, the FFQ used in young children showed a moderate capability to rank the intake frequency of dairy products with a high-fat content and cod liver/fish oil supplements.

Assessing dietary intake in epidemiological studies is a complex undertaking. Valid methods for assessing food intake in children are essential for monitoring dietary habits in early life and studying the implications of dietary factors on health outcomes in childhood and later in life. Methods like food frequency questionnaires (FFQ) allow the collection of data in large samples of individuals. They are easy and relatively inexpensive to administer and can estimate the frequency and sometimes also the amount of the intake of foods and beverages over a defined period of time (1). Other methods, such as 24-h recall and diet record, are usually more expensive and demanding for both the participant and the researcher, and are unrepresentative of the usual intake because the data assessed covers a limited time (1).

It is important to evaluate the dietary assessment methods used in epidemiological studies. Objective biomarkers could be useful for this purpose as their errors are essentially uncorrelated with errors in any dietary assessment method (1). Plasma phospholipid fatty acids are considered good markers of dietary fatty acid intake over a short to medium time period (1–3), show good long-term reproducibility (4, 5), and are considered to be less affected by recent meal intake than plasma triglycerides (1–3). Polyunsaturated fatty acids and odd-numbered saturated fatty acids are considered optimal fatty acid biomarkers as there is a limited or no endogenous synthesis (6, 7). Eicosapentaenoic acid (EPA, 20:5n-3) and docosahexaenoic acid (DHA, 22:6n-3) from oily fish and pentadecanoic acid (15:0) from dairy products are examples of such fatty acids. Several studies in adults have used these biomarkers to evaluate the dietary intake of fish and dairy products or specific fatty acids estimated with an FFQ (8–18). However, among children, there is a lack of studies comparing dietary assessment methods with biomarkers of fatty acids in general (19–23); and, to our knowledge, no studies compare plasma phospholipid pentadecanoic acid, EPA, and DHA, and the frequency of dairy and fish product intake estimated with an FFQ.

In the present study, a simple FFQ was developed to estimate the usual intake frequency of common foods and beverages. The objective was to evaluate the suitability of the FFQ to rank young children according to their intake of dairy and fish products by comparing food frequency estimates to the plasma phospholipid fatty acids pentadecanoic acid, EPA, and DHA.

## Methods

### Subjects and design

**Fig. 1:**
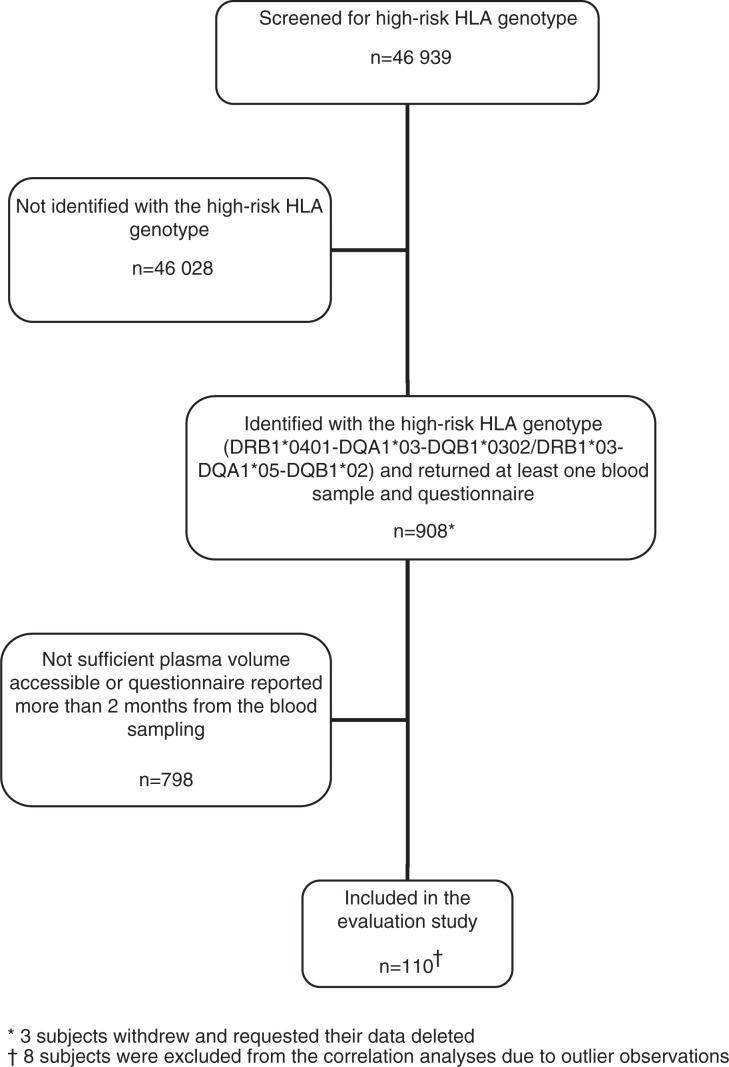
Flow-chart showing ‘Environmental Triggers of Type 1 Diabetes Study’ (MIDIA) participants included in the evaluation study.

Cross-sectional data for the present study were derived from the ‘Environmental Triggers of Type 1 Diabetes Study’ (MIDIA), a prospective ongoing cohort study conducted by the Norwegian Institute of Public Health. The MIDIA study is described in more detail elsewhere (24). Briefly, nearly 50,000 infants from the general population were screened, and 1,003 infants carrying the HLA genotype DRB1*04:01-DQA1*03-DQB1*03:02/DRB1*03-DQA1*05-DQB1*02, conferring the highest risk of type 1 diabetes, were identified during the inclusion period (2001–2007). Questionnaires were filled out by the parents if the child was 3, 6, 9, or 12 months of age, and repeated annually thereafter. Blood samples were obtained from children at the same intervals. In total, 908 subjects (Fig. 1) returned at least one blood sample and questionnaire. The blood samples were sent to the Norwegian Institute of Public Health by ordinary mail and stored at −80°C until further use.

Out of the 908 subjects, 292 subjects were invited to a dietary project, in which 110 subjects were included in this evaluation study in January 2013 (Fig. 1). To be included, the FFQ had to be answered within 2 months of the date of blood sampling, and the volume of plasma accessible from each subject had to be sufficient for the determination of plasma phospholipid fatty acids. Five of the subjects included in the study had blood autoantibodies showing islet autoimmunity, but none had developed type 1 diabetes.

### The food frequency questionnaire

The questionnaire was not quantitative and included questions about the dietary habits of the mother and the child. In the present study, the dietary information about the child's intake frequency of fish and dairy products has been used. This information was derived from three main questions included in the questionnaires used for all age levels, which concerned the intake frequency of (1) oily fish, other fish, fish spread, white cheese, brown cheese, yoghurt, and dairy ice cream; (2) whole fat milk, semi-skimmed milk, skimmed milk, chocolate milk, and yoghurt drink; and (3) cod liver/fish oil supplements (hereinafter referred to as cod liver oil).

### The quantitative determination of fatty acids in the phospholipid fraction of human plasma

An analysis of plasma phospholipid fatty acids was performed at a commercial laboratory in Oslo, Norway (AS Vitas). Plasma stored at −80°C were thawed overnight at 4°C and vortexed for 5 sec. Dichloromethane/methanol was added to 100-µL plasma and 100-µL internal standard (1,2 diheptadecaonyl-*sn*-glycero-3-phosphatidylcholin). After shaking and centrifugation, the supernatants were transferred to new glasses and washed in a 0.9% NaCl solution. The lower phase was transferred to solid-phase extraction columns. Neutral lipids were washed out with dichloromethane/isopropanol and methyl tertiary butyl ether/formic acid. Phospholipids were eluted with methanol. After evaporation to dryness in a vacuum centrifuge, phospholipids were transmethylated with sodium methoxide and fatty acid methyl esters were extracted to hexane before gas chromatography analysis. Analysis was performed on a 7890A Gas Chromatography system with a split/split less injector, a 7683B automatic liquid sampler, and flame ionization detection (Agilent Technologies, Palo Alto, CA). Separation was performed on an SP 2380 (30 m×0.22 mm i.d.×0.25-µm film thickness) column (Supelco, Inc., Bellefonte, PA). The coefficient of variation was 4% for the main fatty acids and 6% for EPA and DHA (AS Vitas, Oslo). Plasma phospholipid fatty acids were expressed as µg/mL and in weight percentage.

### Data analysis

Intake frequency of food items (oily fish, other fish, fish spread, cod liver oil, whole fat milk, semi-skimmed milk, 0.7% fat milk, skimmed milk, chocolate milk, white cheese, brown cheese, yoghurt, yoghurt drink, and dairy ice cream) was reported as frequency per week and recalculated into frequency per day. In addition, different food items were combined into groups, which were *Cheese* (white cheese and brown cheese), *Total milk* (whole fat milk, semi-skimmed milk, 0.7% fat milk, skimmed milk, and chocolate milk), *Total milk/yoghurt* (total milk, yoghurt, and yoghurt drink), *Total fat dairy* (cheese, whole fat milk, yoghurt, and dairy ice cream), *Total dairy* (cheese, total milk/yoghurt, and dairy ice cream), *Total oily fish* (oily fish and fish spread), *Total fish* (other fish and total oily fish), *Cod liver oil/total oily fish*, and *Cod liver oil/total fish*.

Intake frequency estimates and plasma phospholipid acids were presented as mean values and SD, and median values and interquartile range (IQR), since not all of the data were normally distributed. To check for potential outliers, the data were visualized with box and scatter plots, and observations deemed to be outliers were removed from the main analysis. The suitability of the FFQ for ranking participants was assessed by conducting a Spearman correlation analysis and by investigating whether participants are classified into the same quartiles of distribution, based on the reported frequency of dairy product intake and plasma phospholipid pentadecanoic acid, and the frequency of fish product intake and plasma phospholipid EPA and DHA, respectively. The correlation coefficients were calculated for both µg/mL and weight percentage of plasma phospholipid fatty acids, and presented with 95% CI based on 1,000 bootstrap samples. Individuals classified into the same quartile, based on intake frequency and concentration of plasma phospholipids, were defined as ‘correctly classified’, and individuals classified into opposite quartiles were defined as ‘grossly misclassified’. The Spearman correlation analysis of the intake frequency of total oily fish and the plasma phospholipid DHA concentration was also conducted for the subpopulations that consumed or did not consume cod liver oil. Due to concerns regarding the use of pentadecanoic acid as a biomarker in populations with a high fish consumption (25), an additional Spearman correlation analysis of the frequency of fish product intake and plasma phospholipid pentadecanoic acid was conducted. Separate analyses of those who filled in the FFQ before and after the date of blood sampling and an analysis excluding the children with islet autoimmunity were also performed. Statistical analyses were performed with the Statistical Package for Social Sciences statistical software package version 20.0 (SPSS, Inc., Chicago, IL) and STATA version 13 (Statacorp, TX). The chosen level of statistical significance was 5%. Based on previous studies in adults, a correlation of *r*=0.30 was expected. With a significance level of 5% (*α*=0.05) and power 80% (*β*=0.20), the required sample size was estimated to be *n*=85.

## Results

The study included children (*n*=110, 59 boys and 51 girls) with a mean age of 5.6 years (SD 1.6) ranging from 3 to 10 years of age. The median frequency intake of total dairy products was 1.9 times/day and 1.1 times/day for total fat dairy products. The median frequency intake of total fish was 0.6 times/day, and cod liver oil was consumed with a median frequency intake of 0.1 times/day (Table 1). Cod liver oil was consumed by 55.5% of the children (*n*=61). Of the measured plasma phospholipid fatty acids, DHA was found to have the highest mean plasma concentration with 55.3 µg/mL (Table 2). In total, eight observations were considered outliers and excluded in the subsequent analyses.

**Table 1:** The mean (SD) and median (IQR) frequency of dairy and fish product intake estimated with a food frequency questionnaire (*n*=110)^a^

	Frequency (Times per Day)			
	
Food Items/Groups	Mean	SD	Median	IQR
White cheese	0.56	0.55	0.29	0.42
Brown cheese	0.32	0.30	0.29	0.64
Whole fat milk	0.02	0.08	0.00	0.00
Semi-skimmed milk	0.38	0.32	0.29	0.71
Yoghurt	0.21	0.23	0.07	0.22
Cheese	0.89	0.68	0.71	0.77
Total milk	0.60	0.38	0.71	0.49
Total milk/yoghurt	0.89	0.49	0.85	0.58
Total fat dairy	1.30	0.76	1.14	0.81
Total dairy	1.97	0.88	1.92	1.25
Total oily fish	0.38	0.38	0.29	0.44
Other fish	0.23	0.12	0.29	0.22
Total fish	0.61	0.41	0.58	0.32
Cod liver oil	0.35	0.42	0.07	0.64
Cod liver oil/total oily fish	0.73	0.62	0.58	0.95
Cod liver oil/total fish	0.96	0.64	0.82	1.00

a All observations were included.

**Table 2:** The mean (SD) and median (IQR) concentration and weight percentage of plasma phospholipid pentadecanoic acid, EPA, and DHA (*n*=110)^a^

Plasma Phospholipids	Mean (µg/mL)	SD	Mean Weight Percentage	SD	Median (µg/mL)	IQR	Median Weight Percentage	IQR
Pentadecanoic acid	2.20	0.46	0.24	0.04	2.16	0.65	0.23	0.05
EPA	15.8	10.8	1.69	1.07	11.6	10.2	1.30	1.18
DHA	55.3	17.3	5.95	1.71	52.1	20.7	5.77	2.10

a All observations were included.

### Spearman correlation analysis

**Fig. 2:**
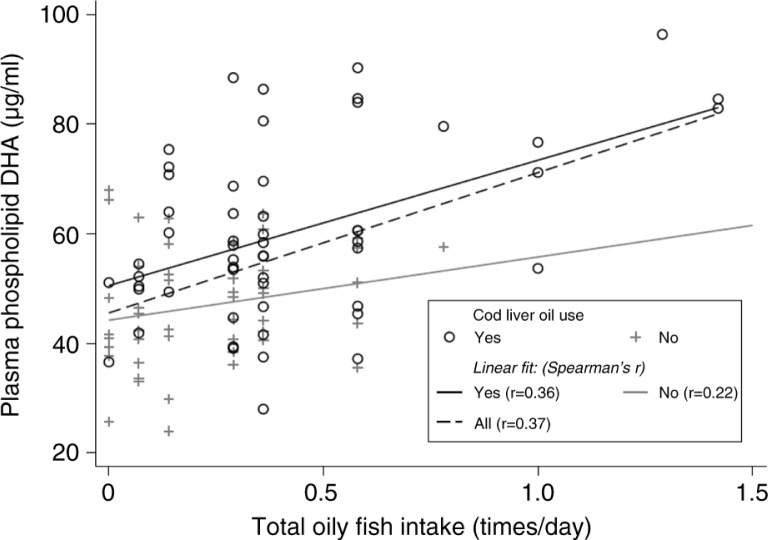
Intake frequency of total oily fish and plasma phospholipid docosahexaenoic acid (DHA) concentration (µg/mL). Distribution of the intake frequency of total oily fish estimated with a food frequency questionnaire (FFQ) and the plasma phospholipid DHA (µg/mL), and Spearman's correlation coefficients for the whole sample (*n*=102), and subpopulations that used cod liver oil (*n*=55) and those that did not (*n*=47). In total, 8 observations were considered outliers and are not shown nor used in the correlation analyses.

Using absolute values (µg/mL) of plasma phospholipid fatty acids, significant correlations (*p*≤0.01) were found between pentadecanoic acid and the intake frequency of total dairy products (*r*=0.29), and total fat dairy products (*r*=0.39) and cheese products (*r*=0.36). There was no significant correlation between pentadecanoic acid and the intake frequency of total milk and yoghurt (Table 3). Significant correlations were observed between the intake frequency of total oily fish and EPA and DHA (*r*=0.26 and 0.37, respectively). Similarly, cod liver oil was significantly correlated with the long chained omega-3 fatty acids (*r*=0.47 for EPA and *r*=0.50 DHA). No significant correlations were observed for the food group other fish (Table 4). The correlation between the intake frequency of total oily fish and DHA was lower for the subpopulation that did not consume cod liver oil (*r*=0.22), than for the group that did (*r*=0.36) (Fig. 2). There was no significant correlation between pentadecanoic acid and the intake frequency of total oily fish (*r*=0.04) or cod liver oil (*r*= − 0.07). Using the relative distribution of fatty acids instead of absolute values in the analysis did not give any major differences in the Spearman's rank correlation coefficients (Tables 3 and 4).

**Table 3:** Spearman's rank correlation coefficients (95% CI^a^) between the frequency of dairy product intake estimated with a food frequency questionnaire (FFQ) and the µg/mL and weight percentage of plasma phospholipid pentadecanoic acid (*n*=102^b^)

	Pentadecanoic Acid	
	
Dairy Product	µg/mL	Weight Percentage
Cheese	0.36 (0.18, 0.52)*	0.39 (0.21, 0.54)*
Whole fat milk	0.12 (−0.11, 0.34)	0.15 (−0.07, 0.33)
Total milk	0.08 (−0.12, 0.27)	0.08 (−0.11, 0.27)
Total milk/yoghurt	0.07 (−0.13, 0.27)	0.09 (−0.12, 0.28)
Total fat dairy	0.39 (0.20, 0.56)*	0.43 (0.26, 0.58)*
Total dairy	0.29 (0.11, 0.47)*	0.31 (0.11, 0.48)*

a Presented with 95% CI based on 1,000 bootstrap samples.

b Eight observations were considered as outliers and excluded from the analysis.

* *P*≤0.01.

**Table 4:** Spearman's rank correlation coefficients (95% CI^a^) between the frequency of fish product intake estimated with a food frequency questionnaire (FFQ) and the µg/mL and weight percentage of plasma phospholipid EPA and DHA (*n*=102^b^)

	EPA	DHA	EPA	DHA
	
Fish product	µg/mL	µg/mL	Weight Percentage	Weight Percentage
Total oily fish	0.26 (0.07, 0.43)*	0.37 (0.18, 0.54)*	0.28 (0.10, 0.45)*	0.40 (0.23, 0.55)*
Other fish	0.07 (−0.11, 0.27)	−0.12 (−0.31, 0.06)	0.07 (−0.11, 0.25)	−0.14 (−0.33, 0.05)
Total fish	0.25 (0.06, 0.42)*	0.29 (0.09, 0.47)*	0.27 (0.08, 0.42)*	0.30 (0.10, 0.47)*
Cod liver oil	0.47 (0.30, 0.61)*	0.50 (0.31, 0.64)*	0.46 (0.28, 0.61)*	0.46 (0.27, 0.61)*
Cod liver oil/total oily fish	0.48 (0.29, 0.62)*	0.51 (0.32, 0.66)*	0.47 (0.30, 0.63)*	0.49 (0.31, 0.64)*
Cod liver oil/total fish	0.46 (0.27, 0.62)*	0.45 (0.26, 0.62)*	0.45 (0.26, 0.60)*	0.43 (0.23, 0.59)*

a Presented with 95% CI based on 1,000 bootstrap samples.

b *n*=8 observations were considered outliers and excluded from the analysis.

* *P*≤0.01.

In comparison to the date of blood sampling, the FFQ was completed at the same date or up to 2 months after for 63.6% (*n*=70) of the participating children, and until 2 months before for 36.4% (*n*=40). A separate analysis showed that there were no major differences between the correlation coefficients of those who filled in the FFQ after or before the blood sampling (data not shown).

Five of the children had blood autoantibodies showing islet autoimmunity, but none had developed type 1 diabetes. An analysis excluding the children with islet autoimmunity (*n*=5), showed unchanged results (data not shown).

### Quartiles of distribution

Based on the intake frequency of dairy and fish products and the concentration of plasma phospholipid pentadecanoic acid, EPA, and DHA, the proportion of subjects appearing in the same quartile varied from 22.6% for the food group, other fish, and DHA, to 42.2% for cod liver oil/total fish and DHA (Table 5). The percentage of subjects grossly misclassified varied from 14.7% for total milk and pentadecanoic acid to 3.9% for cod liver oil/total fish and cod liver oil/total oily fish and EPA and DHA, cod liver oil and DHA, and total fat dairy and pentadecanoic acid (Table 5).

**Table 5:** Cross-classification of subjects by quartiles of the frequency of dairy and fish product intake estimated with a food frequency questionnaire (FFQ) and the µg/mL of plasma phospholipid pentadecanoic acid, EPA, and DHA (*n*=102^a^)

	Correctly classified (%)	Correct plus adjacent quartiles (%)	Grossly misclassified^b^ (%)
Dairy intake and pentadecanoic acid			
Cheese	38.2	73.5	4.9
Total milk	30.3	64.5	14.7
Total milk/yoghurt	27.4	65.6	11.8
Total fat dairy	31.4	75.6	3.9
Total dairy	30.4	70.7	5.9
Fish intake and EPA			
Total oily fish	31.4	71.6	5.8
Other fish	25.5	67.7	5.9
Total fish	29.4	69.4	5.9
Cod liver oil	38.1	72.4	5.9
Cod liver oil/total oily fish	42.1	81.3	3.9
Cod liver oil/total fish	41.1	82.2	3.9
Fish intake and DHA			
Total oily fish	33.3	76.4	4.0
Other fish	22.6	60.8	8.8
Total fish	32.3	74.5	6.9
Cod liver oil	41.1	76.4	3.9
Cod liver oil/total oily fish	42.1	80.4	3.9
Cod liver oil/total fish	42.2	82.4	3.9

a Eight observations were considered as outliers and excluded from the analysis.

b Classified into opposite quartiles.

## Discussion

In the present study, the reported frequency of high-fat dairy product intake was weakly to moderately correlated with plasma phospholipid pentadecanoic acid. The reported intake frequency of total oily fish was weakly correlated, and cod liver oil was moderately to strongly correlated with the long-chain omega 3 fatty acids EPA and DHA.

A strength of the study is that we could choose blood samples close to the time period of the completion of the questionnaire. As plasma phospholipid fatty acids are considered to reflect dietary fatty acid intake over a short to medium time period (1–3), one could assume that the most appropriate design would be to study those who filled in the FFQ in the time period from 2 months before and up to the date of blood sampling. However, a separate analysis showed that there were no major differences between the correlation coefficients of those who reported the FFQ at the date of blood sampling or up to 2 months after and those who reported the FFQ until 2 months before the blood sampling.

We have studied children genetically susceptible to type 1 diabetes, and the results are not necessarily generalizable to the whole population, but to the study population. Nevertheless, it is unlikely that the specific HLA genotype of these children would directly influence their lipid concentrations. In our study, only five children had developed islet autoimmunity, and excluding these did not influence the conclusions. Participation in the study might select for a perceived healthier diet, and there was a higher percentage of cod liver oil users in our study compared to those of a Norwegian national survey among 4-, 9-, and 13-year-olds (26, 27).

It could be considered a limitation that we do not have multiple measures of the food intake or another dietary assessment method, such as 24-h recall or diet record, to compare with the methods we used. However, these self-reporting methods share some of the same sources of error that occur in the reporting of any FFQ for which a biomarker is uncorrelated to errors in any dietary questionnaire (1). A limitation to the study is that there were no specific restrictions on diet in relation to the sampling of blood. The plasma phospholipid fraction could be influenced by a recent meal, although less than the plasma triglyceride fraction would be affected (1–3).

Pentadecanoic acid has been used as a biomarker for the dairy intake in several studies, but since this fatty acid is also present at low amounts in fish, some concerns have been raised regarding its use as a biomarker in populations with a high fish consumption (25). In our population, the intake of both fat dairy and fish products was relatively high, and we found no correlation between pentadecanoic acid and fish products.

Our results in children are in line with those from studies in adults that have correlated the fish intake estimated from FFQ with plasma phospholipid EPA and DHA, with correlations ranging from 0.1 to 0.3 for total fish and 0.4 to 0.5 for cod liver oil (11–13). One study did not report correlation coefficients but showed increased plasma phospholipid EPA and DHA with an increased intake of fish, and higher levels of plasma phospholipid EPA and DHA in people taking cod liver and fish oil supplements (13). In concurrence with our results, similar correlations for absolute and relative measures of plasma phospholipid EPA and DHA have previously been shown in adults (11). Studies in the adult population mainly show significant correlations between the reported intake of fat dairy products or total diary fat and pentadecanoic acid in different blood fractions but not for low-fat dairy products (8–10).

In line with our results, a study on Finnish children, adolescents, and young adults aged 9–24 years (*n*=759) showed significant correlations between the intake of fish reported by 48-h recall and serum phospholipid EPA and DHA, with correlation coefficients of *r*=0.42 and 0.28, respectively (20). A recent study among Finnish children aged 6–8 years reported a significant correlation of *r*=0.15 between the intake of fish reported by 3-day dietary records and plasma phospholipid DHA, but not for EPA (21). A significant correlation of *r*=0.22 between the intake of fatty milk and pentadecanoic acid was reported, but not for the intake of cheese (21). A Finnish study among 1- to 3-year-old toddlers found a positive correlation between the intake of fatty milk reported by 3-day dietary records and pentadecanoic acid in whole serum, but found no consistent correlation between the intake of fish and EPA or DHA (22). Neither of these studies among children analysed the intake of oily and other fish separately, and the authors state that the frequency intake of fish was low, which may affect the correlations. As for dairy, this discrepancy may be due to the low frequency intake of fatty milk and the relatively high intake of cheese reported in our study. Although we did not differentiate between regular (25–40% fat) and low-fat cheese (15–20% fat) in the FFQ, these products are generally high in fat content. An American study among young children, 1–11 years of age with high-risk of developing type 1 diabetes, found a positive correlation of *r*=0.42 between the estimated intake of total marine fatty acids reported by FFQ, and EPA and DHA in the erythrocyte membrane (23). However, they did not investigate the frequency intake of specific food groups.

By classifying individuals into quartiles, we expect, by chance, 25.0% to fall in the same quartile and 12.5% in the other quartiles (12). The FFQ was, to a large extent, able to classify individuals into the same quartile as the relevant biomarker. A higher percentage of correct classification and a lower percentage of misclassification of individuals were found for the intake frequency of dairy products with a high-fat content, compared to dairy products with a low-fat content. The findings from the cross-classification analyses correspond well with the correlations between the reported frequency of dairy product intake and plasma phospholipid pentadecanoic acid. As for dairy products, a higher percentage of correct classification and a lower percentage of misclassification of individuals was found for the intake frequency of fish products high in fat content, compared to fish with a lower fat content. This is in line with the correlations between the reported frequency of fish product intake and plasma phospholipid EPA and DHA.

Of all dairy products, cheese had the strongest correlation with the concentration of plasma pentadecanoic acid. The moderate correlation between the intake frequency of total fat dairy and pentadecanoic acid was mainly explained by the contribution from cheese. The correlation between total oily fish and DHA, stronger than for total oily fish and EPA, reflects the relative content of these marine fatty acids in oily fish. The intake of other fish did not correlate with either EPA or DHA, which could be explained by a lower content of marine fatty acids in lean fish and fish products. There was a lower correlation between total oily fish and DHA for the subpopulation of those who did not use cod liver oil than for those who did, indicating that the correlation between total oily fish and DHA could in part be explained by the use of cod liver oil. However, these data have to be interpreted with caution because the number of participants was lower in the subpopulation analyses (*n*=47 and *n*=55, respectively), and, moreover, a majority of the children who consumed oily fish most frequently also consumed cod liver oil. Of all food items, cod liver oil had the strongest correlation with the concentration of plasma phospholipid fatty acids (*r*=0.47 for EPA and *r*=0.50 for DHA). The similar correlations could be explained by the high and more equally distributed content of EPA and DHA in cod liver oil compared to oily fish.

In conclusion, the present study suggests that when using the plasma phospholipid fatty acids pentadecanoic acid, EPA, and DHA as biomarkers, the FFQ used in young children showed a moderate capability to rank the intake frequency of dairy products with a high-fat content and cod liver oil.
